# Patient Satisfaction With Primary Healthcare Services in Al-Ahsa, Saudi Arabia

**DOI:** 10.7759/cureus.31478

**Published:** 2022-11-14

**Authors:** Suha Albahrani, Hassan Albidy, Norah Alomar, Linah Bin Mutreb, Asma Alkhofi, Zahraa Alsaleh, Jumanah Alessa, Abdullah Alhabrati, Abdullah Alarbash

**Affiliations:** 1 Family Medicine, King Faisal University, Al-Ahsa, SAU; 2 Medical Student, King Faisal University, Al-Ahsa, SAU

**Keywords:** primary healthcare services, family centered care, al-ahsa region, saudi arabia, satisfaction, patient

## Abstract

Background

Patient satisfaction is regarded as a valid and significant indicator of the quality of medical care delivered. Additionally, it has been shown to be linked to better health outcomes. The goal of the study is to assess patients’ overall satisfaction regarding primary healthcare (PHC) services.

Methodology

In this cross‑sectional study conducted in Al-Ahsa, we used the General Practice Assessment Questionnaire and its four subscales with standard cutoffs. A sample of 287 patients was conveniently selected from PHC centers.

Results

A total of 287 patients were included. Patients’ ages ranged from 18 to more than 65 years with a mean age of 32.5 ± 13.9 years old. In total, 177 (61.7%) patients were female, and 225 (78.4%) reviewed the physician for their own problems. A total of 95 (33.1%) patients had chronic health problems. Overall, of the study patients, a total of 38 (13.2%) were highly satisfied regarding provided services, while 45 (15.7%) had a low overall satisfaction level. In general, the mean score (%) of patient satisfaction was 61.9 ± 11.8.

Conclusions

The level of satisfaction with the services provided by PHC centers in Al-Ahsa is moderate. The level of satisfaction concerning some services provided by PHC centers differs significantly according to age, gender, presence of chronic health problems, and employment status.

## Introduction

The first interaction the patient has with the healthcare system is through primary healthcare (PHC) [1]. Patients’ perceived needs and expectations from their healthcare providers do not necessarily align. As a result, one of the key factors influencing healthcare outcomes and service utilization is patients’ experience with their healthcare services [2]. Therefore, assessments of patient satisfaction with PHC visit is crucial for gauging the quality of care as well as for suggesting possible areas for enhancing the PHC scope of practice [3].

A study conducted in over 30 countries suggested a positive impact of continuity of care and patient satisfaction with better health outcomes and fewer emergency department visits among patients frequenting PHC centers [4]. Furthermore, patients’ satisfaction with their health care has often been viewed as a healthcare goal and has been regarded as one of the most significant metrics for assessing the quality of healthcare services [5,6].

Patient interaction with PHC has several domains, including, but not limited to, accessibility, continuity of care, and communication [7]. Several tools have been developed to evaluate patients’ perspectives on their PHC experiences such as the Primary Care Assessment Tool (PCAT), the Components of the Primary Care Index, and the General Practice Assessment Questionnaire (GPAQ) [8-10].

GPAQ has been accepted and widely used for the assessment of satisfaction in PHC internationally [11,12]. It studies various aspects of care, including the accessibility of care, physician and nurse interpersonal skills, communication, and enablement of care [13].

In Saudi Arabia, health care is accessible free of charge for all citizens through governmental PHC centers and general hospitals [14]. As of December 2021, the number of PHC centers within the Kingdom of Saudi Arabia (KSA) was 2,121, 63 of which are in the Al-Ahsa region [15]. With the ongoing PHC reform plans, easier access to care including teleconsultation services through a toll-free number (937) as well as the SEHA Ministry of Health application, 6,500 and 1,500 daily teleconsultations are being offered, respectively [16]. These major reform plans are continuously evolving to meet the overgrowing expectations of the citizens.

Several studies have been conducted in KSA for assessing patient satisfaction assessment, and favorable results have been reported in most [6,17-19]. However, to date, no studies assessing patient satisfaction targeting the Al-Ahsa region have been reported in the literature. The purpose of the study is to assess patients’ overall satisfaction with PHC services and evaluate areas for improvement in our healthcare system within the region.

## Materials and methods

A cross‑sectional study was conducted to assess patients’ overall satisfaction with PHC services. The study was conducted at multiple PHC centers selected conveniently from PHC centers in the Al-Ahsa region. Patients were interviewed by trained data collectors after completing their visit to the PHC center. The study goal was concealed from healthcare providers at the chosen centers to minimize the risk of performance bias after proper administration approval was obtained. The questionnaire was translated into Arabic by consultants who are bilingual speakers, and back translation was performed.

A convenient sampling method was used for data collection. The sample size was calculated using Med Calc statistical software, assuming the area under the receiver operating characteristic (ROC) curve to be 0.80 with an alpha of 0.05 and a power of study of 80.0%. The minimum sample size required for this study was 281 patients. The inclusion criteria included Arabic-speaking patients, caregivers, or caretakers visiting governmental PHC centers in the Al-Ahsa region. We excluded non-Arabic speakers, pediatric patients, and those who could not answer the questionnaire due to hearing difficulties.

After data were extracted, it were revised, coded, and fed to SPSS version 22 (IBM Corp., Armonk, NY, USA). P-values of less than 0.05 were statistically significant. The GPAQ items scores were expressed as a score ranging between 0 and 100, with higher scores representing greater satisfaction. General practice domain measures were the six GPAQ item scores [13]. Data were presented by means and standard deviations for continuous data (outcomes) and frequencies and percentages for categorical data (participant characteristics). A mixed-effects linear regression model based on restricted maximum likelihood estimation and treating General Practitioner (GP) practice as a random effect was used to calculate 95% confidence intervals for the means and to estimate the intracluster correlation coefficient (ICC) for each GPAQ item score. When positive, the ICC can be interpreted as the proportion of the total variation in outcome due to variation between practices. The greater the variation in mean outcome between practices, the greater the ICC [20]. Mixed-effects linear regression models (treating GP practice as a random effect and patient characteristics as fixed effects) were also used to calculate differences in means and 95% confidence intervals for each outcome between each subgroup and the reference group for each participant characteristic, including age group, sex, chronic health problems, and employment status.

The study obtained ethical approval from the Scientific Research Committee (H-05-HS-065). All study participants provided informed consent regarding their voluntary participation. Participants were assured that their information would be kept confidential. Permission was obtained from the administration of PHC centers to conduct the study.

## Results

A total of 287 patients were included in this study. Patients’ ages ranged from 18 to more than 65 years with a mean age of 32.5 ± 13.9 years. Overall, 61.7% of the study participants were females, and around a quarter of the respondents were caregivers of patients. One-third of the study subjects reported chronic health problems. A total of 125 (43.6%) were unemployed or retired, 112 (39%) were employed, and 50 (17.4%) were students (Table 1).

**Table 1:** Personal characteristics of study patients.

Patient characteristics	Number	%
The physician/nurse I saw today was for		
Myself	225	78.4%
My child	56	19.5%
Others	6	2.1%
Age in years		
<44	210	73.2%
45–64	67	23.3%
65+	10	3.5%
Gender		
Male	110	38.3%
Female	177	61.7%
Do you have a long-standing health condition?		
Yes	95	33.1%
No	185	64.5%
I don’t know	7	2.4%
Which of the following best describes you?		
Unemployed/retired	125	43.6%
Employed	112	39.0%
Student	50	17.4%

During their consultation, most respondents reported good feedback about their physician interaction, with more than 80% rating their physicians as either good or very good in assessing their health needs and explaining their condition and management plan, and roughly two-thirds were pleased with their involvement in their own care. Furthermore, 90% of respondents stated they would be happy to be assessed by the same physician again. Similar results were seen with patients’ nursing staff experience, with more than 90% rating their nursing staff as satisfactory to very good in all domains of communication, enablement, and continuity of care.

Regarding the accessibility of care, only 50% of participants reported ease of booking their consultation ahead of time, and similar findings were observed with access to urgent consultation (within the same day). When inquired about the ease of access to a specific healthcare provider in their PHC center, 26% of respondents reported they are usually seen within a couple of days of needing a consult, with 40% rating the time to assessment as good to excellent and 20% rating it as poor or very poor while same-day access to assessment by any healthcare provider was observed with more than 50% of participants. Around one-third of the study participants reported an average waiting time of more than 30 minutes before their consultation, with 27.5% rating their waiting time as poor, and only 7% reporting they were seen in less than five minutes after their registration.

Furthermore, the study revealed that 205 (71.4%) reported waiting times of more than 10 minutes, and only 82 (28.6%) reported waiting times of up to 10 minutes. Regarding the relationship with satisfaction domains, satisfaction for the receptionist score was significantly higher among those who waited for more than 10 minutes than others (83.5 ± 24.1 vs. 77.1 ± 28.1; p = 0.048). On the other hand, overall satisfaction regarding practice was significantly higher among patients who waited for less than 10 minutes than among others (73.7 ± 10.7 vs. 69.4 ± 13.7; p = 0.011) (Table 2).

**Table 2:** GPAQ item scores by patient waiting time. P: independent-samples t-test; * p < 0.05 (significant). CI: confidence interval; SD: standard deviation; GPAQ: General Practice Assessment Questionnaire

Domains	Waiting time				Mean difference	P-value
	≤10 minutes (n = 82)		>10 minutes (n = 205)			
	Mean (95% CI)	SD	Mean (95% CI)	SD		
General practitioner	82.7 (79.7-85.8)	13.9	82.8 (80.8-84.9)	14.3	0.09	0.961
Receptionists	77.1 (70.9-83.3)	28.1	83.5 (80.2-86.9)	24.1	6.40	0.048*
Access to practice	43.7 (38.7-48.7)	22.6	45.6 (42.2-48.9)	24.2	1.85	0.553
Continuity of care	71.0 (64.9-76.8)	27.2	70.0 (66.5-73.3)	24.4	-1.02	0.756
Communication	20.1 (14.2-26.0)	26.9	24.2 (20.4-27.9)	27.5	4.07	0.255
Nursing care	59.4 (53.8-64.9)	25.0	60.1 (56.5-63.6)	25.6	0.65	0.846
Practice overall	73.7 (71.4-76.1)	10.7	69.4 (67.5-71.3)	13.7	-4.34	0.011*
Overall satisfaction	61.1 (58.6-65.4)	11.2	62.2 (59.0-66.3)	12.1	1.1	0.364

Regarding participants’ assessment of their overall experience with PHC in the Al-Ahsa region, around 85.6% rated their experience as satisfactory, and more than 70% reported they would recommend their PHC to relatives and friends. Responses to all items in the GPAQ were properly distributed across response categories. Summary statistics and ICC for each GPAQ item are presented in Table 3. Mean scores for the six GPAQ items ranged from 23.0 (19.9-26.2) for satisfaction with communication to 82.8 (81.1-85.4) for satisfaction with the GP visit.

**Table 3:** GPAQ item scores and ICCs for study participants. CI: confidence interval; SD: standard deviation; GPAQ: General Practice Assessment Questionnaire; ICC: intracluster correlation coefficient

Satisfaction with	n	Mean (95% CI)	SD	Range in mean scores between practices	ICC
GP visit	287	82.8 (81.1-85.4)	14.1	33.4–97.9	0.663
Receptionists	287	81.7 (78.8-84.7)	25.4	0–100	0.125
Access to practice	287	45.1 (42.3-47.8)	23.8	0–100	0.089
Continuity of care	287	70.2 (67.3-73.2)	25.2	0–100	0.119
Communication	287	23.0 (19.9-26.2)	27.4	0–80	0.102
Nursing care	287	59.9 (56.9-62.8)	25.4	0–96.9	0.074
Practice overall	287	70.6 (69.1-72.1)	13	12.5–38.3	0.362

Multivariate regression analysis for each of the GPAQ items showed that when adjusting for other participant characteristics, older patients (those over 65 years) gave lower ratings than younger patients on each of the GPAQ scales except for access to practice, communication, nursing care, and practice overall (Table 4). Female patients gave significantly lower ratings than male patients on communication and nursing care. Patients without chronic health problems showed significantly lower satisfaction with communication than others with chronic diseases. Moreover, employed patients showed significantly higher satisfaction with GP visits than unemployed participants.

**Table 4:** Results of multivariable linear regression analysis for each GPAQ item score by patient characteristics. Figures are differences (95% CI) in mean outcomes between each category and the reference category for each patient factor adjusted for remaining patient factors listed in the table (reference value was designated as zero for calculating differences). Estimates were made using a mixed-effects linear regression model, where GP was treated as a random effect and all other factors were treated as fixed effects. P-values summarize the strength of association between the GPAQ item and the patient characteristic. *: P < 0.05 (significant). Diff: difference; GP: general practitioner; GPAQ: General Practice Assessment Questionnaire; Ref: reference category; CI: confidence interval

Factors	General practitioner			Receptionists			Access to practice			Continuity of care			Communication			Nursing care			Practice overall		
	Diff	95% CI		Difference	95% CI		Diff	95% CI		Diff	95% CI		Diff	95% CI		Diff	95% CI		Diff	95% CI	
Age in years																					
<44	Ref			Ref			Ref			Ref			Ref			Ref			Ref		
45–64	-2.0	-9.7	5.6	-3.7	-22.3	14.9	6.2	-9.0	21.4	-2.7	-18.0	12.7	11.2	-6.4	28.9	12.1	-2.3	26.4	6.2	-1.6	14.0
65+	-5.5	-15.0	4.0	-3.3	-19.4	12.7	6.9	-8.5	22.3	-4.3	-20.8	12.2	12.9	-4.7	30.4	13.1	-3.8	29.9	1.7	-7.0	10.4
P-value	0.033*			0.859			0.801			0.254			0.719			0.714			0.527		
Gender																					
Male	Ref			Ref			Ref			Ref			Ref			Ref			Ref		
Female	-3.2	-6.5	0.2	-1.7	-7.8	4.4	-2.7	-8.4	3.0	2.3	-3.8	8.3	-6.8	-13.3	-0.3	-7.9	-13.9	-1.8	2.5	-0.6	5.6
P-value	0.175			0.485			0.635			0.471			0.001*			0.001*			0.106		
Chronic health problems																					
Yes	Ref			Ref			Ref			Ref			Ref			Ref			Ref		
No	0.5	-3.1	4.0	-0.6	-6.9	5.7	-3.6	-9.5	2.3	0.1	-6.1	6.3	-6.1	-12.8	0.1	-2.1	-8.4	4.2	0.6	-2.7	3.8
P-value	0.937			0.174			0.524			0.996			0.001*			0.157			0.940		
Employment																					
Unemployed/retired	Ref			Ref			Ref			Ref			Ref			Ref			Ref		
Employed	4.6	1.0	8.2	1.9	-4.4	8.2	0.1	-5.9	6.1	-2.1	-8.5	4.4	2.8	-4.0	9.7	2.6	-3.9	9.2	-0.3	-3.5	3.0
P-value	0.002*			0.425			0.748			0.344			0.719			0.718			0.694		

**Figure 1:**
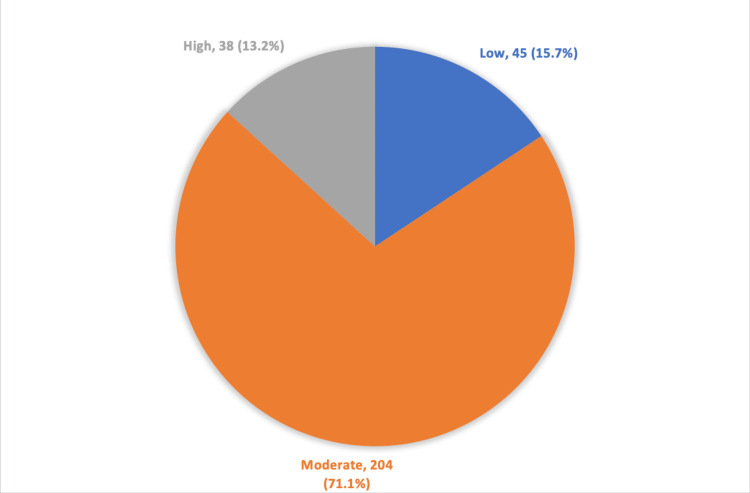
Overall satisfaction of study patients concerning primary healthcare services.

Overall, of the study patients, a total of 38 (13.2%) were highly satisfied regarding provided services, while 45 (15.7%) had a low overall satisfaction level. The overall mean score (%) of satisfaction was 61.9 ± 11.8 (Figure 1).

## Discussion

Patient satisfaction is regarded as an important measure of the quality of care and has been linked to better patient outcomes. We aimed in this study to assess the satisfaction of patients and their caregivers with PHC in the Al-Ahsa region and examine possible areas for improvement in our healthcare system.

Overall satisfaction with PHC was favorable and comparable to other regions within KSA [6,17]. However, a study conducted in Jeddah reported a lower overall satisfaction score (60%). This may be attributed to patients living at a distance from PHC centers and having less knowledge about the presence of a Family Physician (FP) in their nearest PHC centers [21].

Higher satisfaction scores were observed in Kuwait, Lebanon, and Egypt (99.6%, 98%, and 96.6%, respectively). This can be explained by differences in the assessment tool, cultural differences, and the lack of clear healthcare expectations and goal assessment for our study population [22-24].

In Riyadh, one study found the highest rates of satisfaction reported for communication and staff at 72.7% and 73.4%, respectively [4]. In our study, participant satisfaction was the highest regarding GP visits and receptionists (82.8% and 81.7%, respectively), while it was the least concerning communication (23%). Regarding factors related to patients’ satisfaction with medical consultation at PHC centers and participants’ GP visits, they differed significantly with participants’ ages and employment status, with lower satisfaction among retired or unemployed individuals. This is may be explained by unmet higher expectations experienced by these two groups of patients. In addition, regarding participants’ gender, their scores for nursing care differed significantly, with lower satisfaction scores among female participants. Different findings were reported by different studies.

In Abha, one study showed that participants’ total communication scores differed significantly according to their age groups, with lower satisfaction scores among younger participants [1]. Moreover, women reported lower levels of satisfaction than males. This study is in line with a similar study conducted in Jeddah which showed around 40% of the patients were not satisfied by their relationship with the FP due to poor communication [5]. The present study showed that participants’ communication scores differed significantly according to their gender and the presence of chronic health problems, with lower satisfaction scores among female participants and in those without chronic diseases. However, participants’ communication scores did not differ significantly according to their age group or employment status. Lower satisfaction scores were seen in participants without chronic illness in the communication domain of the GPAQ. Patients without known comorbidities requiring frequent assessment and medication refills are less likely to visit PHC frequently and those unfamiliar with the staff or the system are also more likely to be unfamiliar with their current complaints and require longer consultations and expect better communication than those familiar with their illness and physicians.

In a study in Arar, difficulty with obtaining appointments was reported by 23.3% of the participants. Waiting time for more than 30% of our study population was more than 30 minutes which seems to be a common issue in most satisfaction studies conducted in the KSA [17,19,21]. This represents a key area for future policy improvement keeping in mind that despite our best efforts in booking and organizing patients consultations, unpredictable factors including patients with emergency complaints, drop-ins, and patients with complex medical complaints requiring lengthy assessment as well as earlier attendance than scheduled appointment might play a role in waiting time and should be factored in waiting time assessment. Furthermore, a study done in Lebanon reported high overall satisfaction (96.6%) despite the mean waiting time for consultation of 28 minutes, which can be explored in future studies in KSA [23].

Patient satisfaction is becoming a more important factor in the assessment and planning of care. Patient evaluations can also assist physicians in focusing more on the requirements of their patients by letting them know their triumphs and shortcomings. Therefore, to raise healthcare standards, it is essential to frequently perform patient satisfaction surveys across all disciplines. The research might aid in the adaptation of policymakers to the growing rivalry among healthcare providers. Our findings indicate that while there is a moderate level of overall satisfaction with services, there is some dissatisfaction with certain features of these services.

A few limitations of our study include the single assessment, cross‑sectional design, and convenient sampling. Moreover, only a few baseline characteristics were evaluated as predictors of patient satisfaction. We recommend including more predictors that may influence patient satisfaction as well as the assessment of patients’ perception and expectations of physicians of further experimental studies for possible intervention to improve overall satisfaction in KSA.

## Conclusions

The objective of this study is to assess how content Al-Ahsa city patients are with the PHC system. According to our study, patients were overall pleased with their care. It was determined that satisfaction is multifactorial, encompassing patient characteristics and the nature of the service offered. Moreover, no one factor can adequately capture the attitude concerning satisfaction with PHC services. As the service being measured varies, the degree of satisfaction is influenced and altered. Additionally, the characteristics of the individual who will be receiving the service are also an influencing factor. Future research may contribute further to this study by exploring the reason behind the general reduced satisfaction as well as highlighting other reasons for dissatisfaction not outlined in this study.
