# Correction: Patient Satisfaction With Primary Healthcare Services in Al-Ahsa, Saudi Arabia

**DOI:** 10.7759/cureus.c87

**Published:** 2023-01-03

**Authors:** Suha Albahrani, Hassan Albidy, Norah Alomar, Linah Bin Mutreb, Asma Alkhofi, Zahraa Alsaleh, Jumanah Alessa, Abdullah Alhabrati, Abdullah Alarbash

**Affiliations:** 1 Family Medicine, King Faisal University, Al-Ahsa, SAU; 2 Medical Student, King Faisal University, Al-Ahsa, SAU

This article has been corrected at the request of the authors due to the misspelling of two author names. They have been corrected as detailed below. The authors deeply regret that these errors were not caught prior to publication.

'Jumana Alessa' corrected to 'Jumanah Alessa' 'Lena Almotreb' corrected to 'Linah Bin Mutreb'

